# Adriamycin-Induced Nephropathy is Robust in N and Modest in J Substrain of C57BL/6

**DOI:** 10.3389/fcell.2022.924751

**Published:** 2022-06-16

**Authors:** Claire Bryant, Rachel Cianciolo, Rajgopal Govindarajan, Shipra Agrawal

**Affiliations:** ^1^ Center for Clinical and Translational Research, Abigail Wexner Research Institute at Nationwide Children’s Hospital, Columbus, OH, United States; ^2^ Department of Veterinary Biosciences, The Ohio State University, Columbus, OH, United States; ^3^ Niche Diagnostics, LLC, Columbus, OH, United States; ^4^ Division of Pharmaceutics and Pharmacology, College of Pharmacy, The Ohio State University, Columbus, OH, United States; ^5^ Translational Therapeutics, The Ohio State University Comprehensive Cancer Center, Columbus, OH, United States; ^6^ Department of Pediatrics, College of Medicine, The Ohio State University, Columbus, OH, United States

**Keywords:** glomerular disease, focal segment glomerulosclerosis, animal model, adriamycin (ADR), podocyte

## Abstract

Adriamycin (ADR)-induced nephropathy remains the leading model to study human primary focal segmental glomerulosclerosis (FSGS), a common pathway for podocyte damage and glomerular loss of function that leads to chronic kidney disease. However, the use of this model for reverse genetics is limited by historical categorization of C57BL/6 mice as an ADR-resistant strain, which is also the most common genetically modified strain. Additionally, conflicting reports exist utilizing C57BL/6 for ADR-nephrosis due to lack of understanding of substrain differences (J/N) and variability in ADR dosage, timing, and frequency to induce damage. We have undertaken a systematic approach to elucidate the specifics of ADR-nephrosis in C57BL/6 N and J substrains. We induced nephropathy with 2 doses of ADR, and measured albuminuria for 6 weeks and performed histological evaluations. Our findings revealed induction of robust and modest proteinuria in N and J substrains, respectively. The serum creatinine levels were elevated in N, but not J substrain. Both the substrains showed reduction in body weight with N greater than J, although mortality remained at 0% in both substrains. Histological analysis showed worse renal lesions in the N than the J substrain. Podocyte markers synaptopodin, nephrin, podocin, and WT1 were reduced to a greater extent in the N than the J substrain. In summary, we provide the nephrology community with a reproducible mouse model for FSGS, in a strain otherwise assumed to be ADR-resistant and highlight the differences between J and N substrains. This enables future studies, especially concerning genetically manipulated animal models in C57BL/6.

## Introduction

Various animal models have emerged to recapitulate the human forms of nephrotic syndrome to facilitate the discoveries to understand the mechanisms and therapeutic possibilities for glomerular disease (Pippin et al., 2009; Yang et al., 2018). Adriamycin (ADR)-induced nephropathy has been the leading model used to study human primary focal segmental glomerulosclerosis (FSGS), a common pathway for podocyte damage and glomerular loss of function that leads to kidney damage and failure (Wang et al., 2000; Pippin et al., 2009). Commonly used both in rats and mice, the ADR model has been used to study the mechanisms that develop in FSGS and chronic kidney disease (CKD) as it allows for a spatio-temporal evaluation of pathophysiological events. Unfortunately, this powerful model is highly strain-dependent in mice, as some strains are more resistant than others to ADR injections (Kimura et al., 1993; Pippin et al., 2009). Historically, the mouse strains BALB/cJ and 129/SvJ have been deemed to be highly susceptible and C57BL/6 to be resistant to ADR-induced nephrosis, attributed to the *Prmt7* (protein arginine methyltransferase 7) and *Prkdc* (protein kinase, DNA-activated, catalytic polypeptide) gene defects conferring susceptibility to ADR (Zheng et al., 2005; Zheng et al., 2006; Papeta et al., 2010). This has been a major impediment in glomerular disease research as C57BL/6 is very commonly used when generating genetic glomerular disease models and a typical strain used for many knock-out and knock-in models. A recent editorial has briefly suggested that the N, but not the J substrain of C57BL/6 mice can be made modestly susceptible to ADR-induced nephropathy using a single ADR dose administration (Arif et al., 2016), while other reports point out the susceptibility of C57BL/6 without substrain specifications using high or multiple doses of ADR (Lee and Harris, 2011; Hakroush et al., 2014; Sonneveld et al., 2017; Yang et al., 2018). Overall, there are conflicting reports in literature reporting the use of this model of nephrosis in C57BL/6, and they are largely due to: 1) lack of a clear understanding of the substrain differences between J and N in C57BL/6, 2) variability of dosages of ADR administered, and 3) the variability in the timing and frequency of ADR administration. To minimize ambiguities, here, we sought to elucidate the specifics of ADR-induced nephropathy in N and J C57BL/6 substrains. Our findings highlight the differences in susceptibility magnitudes of N and J C57BL/6 substrains to ADR-induced nephropathy and provide the nephrology community with a reproducible mouse model for FSGS, in an otherwise assumed to be an ADR-resistant strain.

## Experimental Procedures

### Animal Studies

**FIGURE 1:**
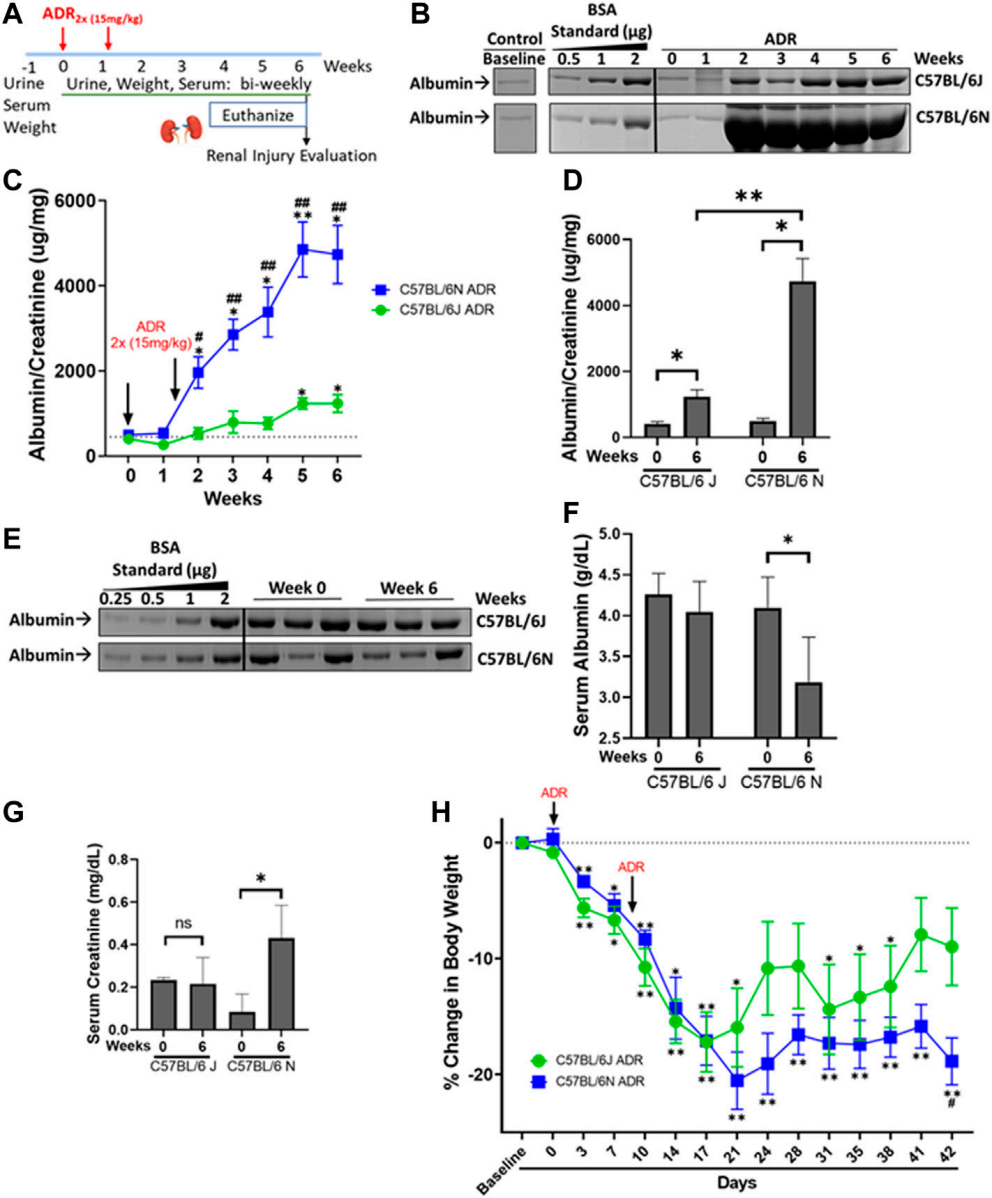
ADR-Induced Proteinuria is Robust in the N and Modest in the J Substrain of C57BL/6 mice. **(A)** C57BL/6 J and N mice were injected with ADR (15 mg/kg/bw, i.v.) twice (Day 0 and Day 9). Urine, serum, and weight were collected throughout the study, and the mice were euthanized 6 weeks after the first injection, on Day 42, at which time the kidneys were harvested. **(B)** Representative urine gels with albumin bands are shown. Equal volumes of urine (5 µl) were analyzed by SDS-PAGE and Coomassie Blue staining. BSA (bovine serum albumin) standards were loaded at determined amounts and used to make a densitometry standard curve to determine albumin concentration. Urine from healthy un-injected mice were used as control. **(C)** Albumin/creatinine ratio was determined and plotted (*p < 0.05, **p < 0.01 vs. baseline of the same substrain, ^#^ *p* < 0.05, ^#^ *p* < 0.01 vs. J substrain at same week, Student *t*-test, *n* = 4/substrain). Dashed line parallel to *X* axis denotes the baseline albuminuria **(D)** Albumin/creatinine ratios plotted from urine samples from Week 0 and Week 6 for J and N substrains (*p < 0.05, **p < 0.01, Student *t*-test, *n* = 4/substrain). **(E)** Representative serum gels with albumin bands shown. Equal volumes of diluted serum (1 µl of 20X dilution) were analyzed by SDS-PAGE and Coomassie Blue staining. BSA (bovine serum albumin) standards were loaded at determined amounts and used to make a densitometry standard curve to determine albumin concentration. **(F)** Albumin values were determined and plotted (*p < 0.05 vs. baseline, Student one tailed *t*-test, *n* = 4/substrain). **(G)** Serum creatinine levels from Week 0 and Week 6 of both J and N substrains are plotted as measured by the enzymatic creatinine test kit (*p < 0.05). **(H)** Decrease in percentage body weight of both J and N substrains of mice at various time points compared to baseline, throughout the study are plotted. (*p < 0.05, **p < 0.01 vs. week 0 of same substrain, #*p* < 0.05 vs. J substrain of same week Student *t*-test, *n* = 4/substrain). Dashed line parallel to *X* axis denotes the baseline starting body weight.

The IACUC at Nationwide Children’s Hospital approved this study. Male C57BL/6J and C57BL/6N mice were purchased at 8 weeks old from Jackson Laboratories (C57BL/6NJ, Strain # 005304, RRID: IMSR_JAX:005304, Common Name: B6N; C57BL/6J, Strain # 000664, RRID: IMSR_JAX:000664, Common Name: B6) and acclimated for 3 days. Both C57BL/6J and C57BL/6N mice were administered Adriamycin (ADR) (Sigma-Aldrich, St. Louis, MO) intravenously (IV) (15 mg/kg) on Day 0 and Day 9 (*n* = 4 each substrain) (Figure 1A). Control male J and N mice between the ages of 6–9 weeks did not receive ADR (*n* = 3/4 each substrain). Spot urine and serum were collected, and body weight recorded at baseline and regular biweekly points throughout the study. All ADR-injected mice experienced weight loss and a few mice (mostly N substrain) exhibited lethargy and dehydration. To minimize the symptoms of loss of appetite and body weight, lethargy, and dehydration, NutraGel (Bio-Serv, Flemington, NJ, United States) and wet chow were given on Days 9 and 29. The mice that exhibited wounds and swelling on their tails due to ADR injection were given buprenorphine and antibiotic ointment on Day 29 for pain alleviation. The mice were sacrificed by exposure to CO_2_ on Day 42 and kidneys were harvested.

### Urinalysis and Serum Chemistry

Urine collected bi-weekly throughout the study was resolved using sodium dodecyl sulfate-polyacrylamide gel electrophoresis (SDS-PAGE). Albumin standards [0.5, 1.0, and 2.0 µg bovine serum albumin (BSA)] were resolved alongside the urine samples on 8% gels, which was then stained with Coomassie Brilliant Blue G-250 (Alfa Aesar, Tewksbury, MA, United States) to visualize bands. Diluted serum was resolved on SDS-PAGE alongside albumin standards as well. The urinary and serum albumin was determined by densitometry from the BSA standard curves using Image J software (National Institutes of Health, Bethesda, MD, United States). Urinary (diluted 10X) and serum (undiluted) creatinine levels were determined using the Enzymatic Creatinine Test Kit (Diazyme, Poway, CA, United States), creatinine standards, and control. Albuminuria was determined and reported by normalizing the urinary albumin values to creatinine of the spot urine samples.

### Histology

Kidney halves showing the cross sections of kidney containing cortex, medulla, and papilla were fixed in 10% buffered formalin for 48 h, followed by 3X PBS washes, and then placed in 70% ethanol for 24 h. They were then processed routinely and embedded in paraffin. The paraffin blocks were sectioned to 4 µm thickness, dried overnight, and baked at 60°C for 1 h. The slides were stained with periodic acid-Schiff method and reviewed by a pathologist blinded to the treatment method. For each animal, 100 glomeruli were assessed for segmental or global sclerosis and evidence of podocyte injury and the tubulointerstitium was semi-quantitively scored.

### Immunofluorescence

Paraffin embedded kidneys were sectioned at 4 µm and deparaffinized with xylene and rehydrated in graded ethanol. Antigen retrieval was performed by boiling in 10 mM sodium citrate for 25 min, followed by washes in PBS-Tween (0.5% Tween-20). After blocking with SuperBlock (Scytek Labs Inc., Logan, UT, United States) for 60 min at 37°C followed by overnight at 4°C, the sections were incubated with primary antibodies [anti-synaptopodin (Santa Cruz Biotechnology, Dallas, TX, United States), anti-nephrin (Proteintech, Rosemont, IL, United States) and anti-WT1 (MilliporeSigma, Burlington, MA, United States)] overnight at 4°C at appropriate dilutions. Sections were washed with 2.5% SuperBlock in PBS-Tween three times, then incubated with secondary antibody (Alexa Fluor 488; Invitrogen, Carlsbad, CA, United States) in SuperBlock. The slides were mounted with Prolong Gold Antifade Mountant (Invitrogen, Carlsbad, CA, United States) and viewed and imaged with equal exposures for each primary antibody with BZ-X700 all-in-one fluorescence microscope (Keyence Inc., Itasca, IL, United States).

### RNA Isolation and Real Time Reverse Transcriptase-Polymerase Chain Reaction

Kidney cortex was isolated and total RNA extracted using the RNeasy Kit (Qiagen, Germantown, MD, United States), according to manufacturer’s instructions. Tissue in lysis buffer was lysed with a stainless-steel disruption bead in a Tissue Lyser (Qiagen, Germantown, MD, United States) for 1 min at 30.0 Hz, and RNA was isolated from the resulting lysate. Yield and purity was determined by measuring the absorbance on a spectrophotometer. 1 μg of RNA was DNase-digested (Invitrogen, Carlsbad, CA, United States), then inactivated with 25 mM EDTA at 65°C for 10 min cDNA was then synthesized by reverse transcription with the iScript cDNA Synthesis Kit (Bio-Rad, Hercules, CA, United States), following manufacturer’s instruction. cDNA was used for qualitative reverse transcription-polymerase chain reaction (qRT-PCR) using primers specific to *Nphs2* (F: ACC​TTT​CCA​TGA​GGT​GGT​AAC, R: CTG​GAT​GGC​TTT​GGA​CAC) normalized to *Rpl6* (F: CTG​ATC​ATC​CTC​ACT​GGG​CG, R: GCG​CAG​AGG​AAC​TCT​GTT​GA). PCR was performed using SYBR green (Bio-Rad, Hercules, CA, United States) on the Applied Biosystems 7500 Real-Time PCR System. PCR conditions were as follows: 95°C for 10 min, 40X (95°C for 15 s, 60°C for 1 min), followed by a melt curve to ensure specific products. The ΔΔCt method was used to analyze the results as described previously (Bryant et al., 2022).

### Statistical Analysis

Statistical analysis was performed using unpaired Student’s *t*-test and paired Student’s *t*-test using the GraphPad Prism software version 8.2.0 for Windows (GraphPad Software, San Diego, CA, United States), as applicable to the dataset.

## Results

### ADR-Induced Proteinuria is Robust in the N and Modest in the J Substrain of C57BL/6 Mice

To determine and compare the extent of susceptibility of C57BL/6 mouse substrains J and N to ADR-induced nephropathy, two doses of ADR injections (15 mg/kg) were administered 10 days apart (Figure 1A). Albuminuria appeared in both substrains during week 2 and continued to increase through week 6. While C57BL/6 J mice showed a modest induction of proteinuria, the N substrain showed massive proteinuria after the second dose of ADR (Figures 1B,C). The N substrain showed an increase in albuminuria starting at week 2 with a robust 9.5-fold increase from baseline at week 6 (4737 ± 682.9 mg/mg vs. 498.2 ± 84.71 mg/mg; *p* = 0.0113) (Figures 1C,D). The J substrain showed a modest increase starting at week 5, which was 3-fold from baseline at week 6 (1237 ± 208.2 mg/mg vs. 405.2 ± 74.23 mg/mg, *p* = 0.0170) (Figures 1C,D). Additionally, albuminuria was consistently higher in the N vs. J substrain for any week between weeks 2–6 (4737 ± 682.9 mg/mg vs. 1237 ± 208.2 mg/mg; 3.8-fold; *p* = 0.0027) (Figure 1C). Control mice, not injected with ADR had similar urine albumin/creatinine amounts as baseline or Day 0 samples from ADR-treated mice, for both J and N substrains (Figure 1B). Dehydration and lethargy experienced in some mice were minimized by providing them with wet chow and NutraGel on days 9 and 29, which likely led to the relatively diluted urine and apparently lowered albumin at week 6 compared to week 2 (Figure 1B). However, normalization of urinary albumin to urinary creatinine values still showed the continuous rise in albuminuria in these mice (Figure 1C). Furthermore, assessment of serum albumin levels showed a decrease in ADR-injected mice at week 6 as compared to baseline/week 0 in the N substrain (3.19 ± 0.5 g/dl vs. 4.1 ± 0.4 g/dl; *p* = 0.05). The decrease in the J substrain was milder, although a similar trend of decrease in serum albumin was observed with ADR-nephrosis at week 6 compared to baseline/week 0 (4.04 ± 0.4 g/dl vs. 4.26 ± 0.2 g/dl) (Figures 1E,F).

### ADR-Induced Nephropathy Results in Elevated Serum Creatinine in the N Substrain Only and Decreased Body Weight in Both the Substrains

Serum creatinine level is an indicator of glomerular filtration rate and progression of nephropathy to chronic stage. Serum creatinine was elevated in the N substrain of C57BL/6 mice, where it increased ∼5-fold at week 6 compared to baseline/week 0 (0.43 ± 0.15 mg/mg vs. 0.08 ± 0.08 mg/mg, *p* = 0.0441) (Figure 1G). However, serum creatinine did not change in the J substrain at the observed time points (Figure 1G). Weight loss was observed in both substrains, with N to a greater extent by week 6 (Figure 1H). Body weight decrease compared to baseline started during week 1 for both substrains, with the weights increasing a non-significant amount around week 4 when the mice were given pain medications and wet chow to reduce dehydration but decreasing again by the next week. Final day weights showed higher body weight decrease in the N than the J substrain (Figure 1H).

### Glomerular and Tubular Histological Alterations Are More Prominent in N vs. J Substrain, and Are Correlative With Albuminuria

**FIGURE 2:**
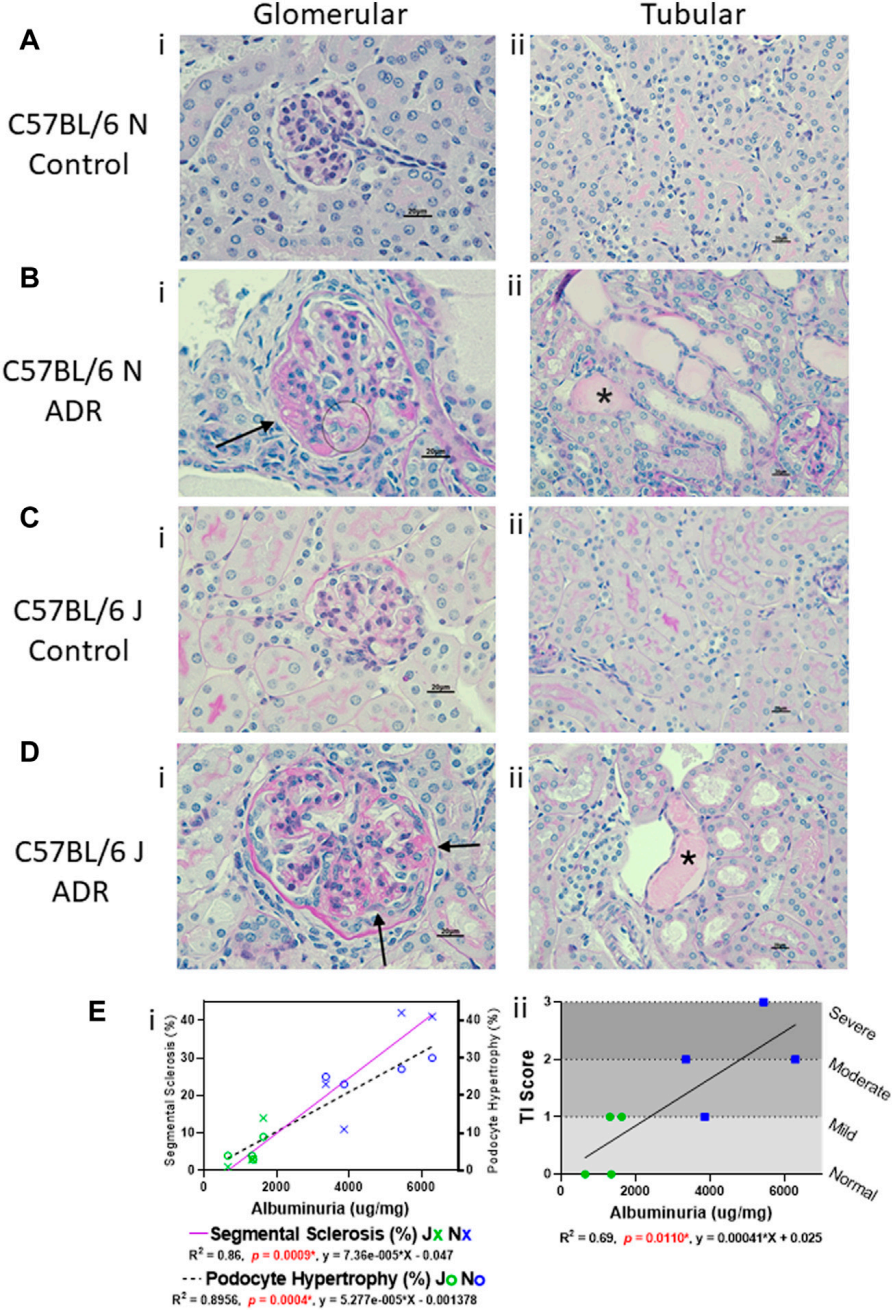
Glomerular and Tubular Histological Alterations are more Prominent in N vs. J Substrain, and are Correlative with Albuminuria. **(A–D)** Histologic evaluation of kidneys stained with periodic acid-Schiff method (scale bar = 20 μm). Representative images showing **(i)** glomerular and **(ii)** tubular histology from **(A,B)** C57BL/6 N control and ADR-injected mice, and **(C,D)**, C57BL/6 J control and ADR-injected mice. **(Bi and Di)** Glomerular damage is indicated with an arrow for a sclerotic segment and circle for hypertrophied podocytes that contain protein reabsorption droplets. **(Bii and Dii)** Tubular protein casts are indicated with asterisks (*). **(E i)** Correlation of glomerular segmental sclerosis and podocyte hypertrophy with albuminuria in J and N ADR-injected mice. Segmental sclerosis is indicated with a purple solid line, and the data points are marked as X’s (Blue = N, Green = J). Podocyte hypertrophy is indicated with a dashed line, and the data points are marked as circles (Blue = N, Green = J). **(E ii)** Correlation of tubular injury (TI) scores with albuminuria in J and N ADR-injected mice (Blue = N, Green = J). The tubular injury was scored subjectively with 0 = normal, 1 = mild, 2 = moderate, 3 = severe.

Histological evaluation and morphometric quantification showed that the N substrain mice injected with ADR displayed segmental sclerosis in some glomeruli, as well as hypertrophied podocytes and tubular degeneration with protein casts, interstitial fibrosis, and tubular atrophy (Figure 2; Table 1). The J substrain had fewer glomeruli with segmental glomerulosclerosis and podocyte injury compared to the N substrain (Figure 2). Segmental sclerosis was characterized by effacement of peripheral capillary lumens by extracellular matrix and these segments were often adhered to Bowman’s capsules. Other lesions which have been reported in models of FSGS (e.g., mesangiolysis, crescents and hypercellularity) were not observed in either substrain. The tubulointerstitial scores were higher in the N substrain with larger portions of the renal parenchyma exhibiting tubular dilation and protein casts. Lesions of acute tubular necrosis and tubular epithelial cell cytoplasmic protein droplets were not observed in either substrain. Neither N nor J control groups had any of the aforementioned pathological lesions (Figure 2; Table 1).

**TABLE 1:** Histological Scoring of ADR-Induced Nephropathy in both N and J Substrains.

ID	Glomerular					Tubular	
	% Segmental sclerosis	Score	% Global sclerosis	% Podocyte hypertrophy	% Histologically normal	Qualitative assessment of damage	Score
C57BL/6 N Control							
#15	0	0	0	1	99	Normal	0
#16	0	0	0	3	97	Normal	0
#17	0	0	0	2	98	Normal	0
C57BL/6 N ADR							
#5	42	3	1	27	30	Severe multifocal protein casts; some regions have tubular basophilia	3
#6	23	2	1	25	51	Moderate multifocal protein casts; some regions have tubular basophilia	2
#7	41	3	1	30	28	Moderate multifocal protein casts; some regions have tubular basophilia	2
#8	11	1	1	23	65	Mild multifocal protein casts; some regions have tubular basophilia	1
C57BL/6 J Control							
#9	0	0	0	8	92	Normal	0
#10	0	0	0	5	95	Normal	0
#11	1	0	0	6	93	Normal	0
#12	0	0	0	9	91	Normal	0
C57BL/6 J ADR							
#1	3	0	0	4	93	Mild multifocal tubular basophilia with protein casts	1
#2	3	0	0	3	94	Normal	0
#3	14	1	0	9	77	Mild multifocal protein casts	1
#4	1	0	0	4	95	Normal	0

Podocyte lesions included cytoplasmic vacuolation, protein reabsorption droplets and synechiae. The tubulointerstitium was semi-quantitively scored for tubular dilation, epithelial basophilia and tubular protein casts. The severity score was based on the portion of the tissue section that was affected. Normal samples received a score of 0. Mildly affected samples had approximately 1/3 of their parenchyma with lesions and they received a score of 1; moderate scores (2) were given when between 1/3 and 2/3 of the renal parenchyma had lesions; severely affected kidneys (3) had more than 2/3 of their parenchyma with lesions. Kidney scoring sheet of 100 glomeruli per sample. Scoring for sclerosis is based on <5% = 0, 5%–20% = 1, 20%–40% = 2, and >40% = 3 (Wharram et al., 2005). Tubular injury was assessed subjectively and the scoring system is 0 = normal, 1-mild, 2 = moderate, 3 = severe.

### Albuminuria is Associated With Podocyte Injury in N and J Substrains With Adriamycin-Induced Nephropathy

**FIGURE 3:**
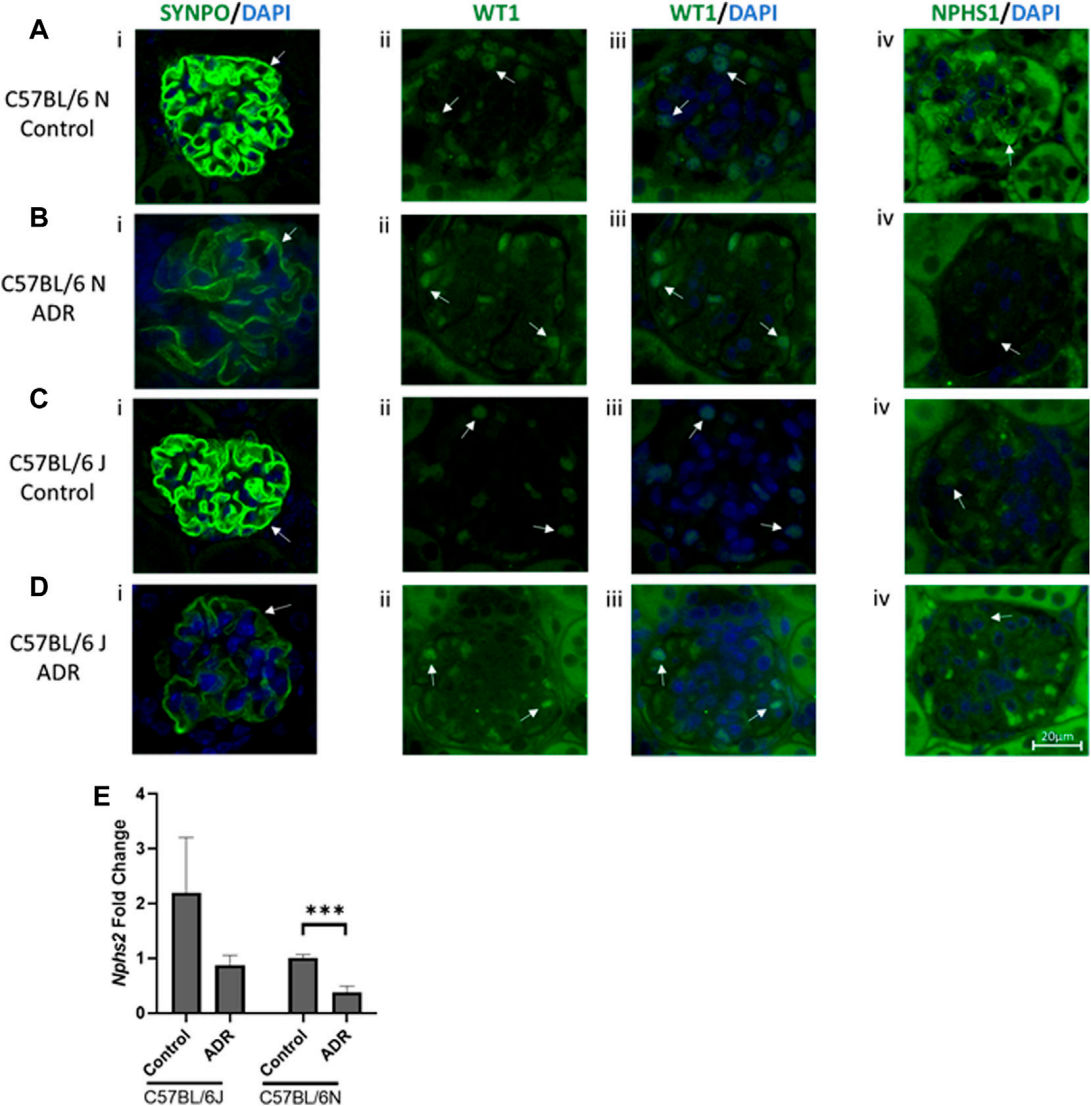
Albuminuria is Associated with Podocyte Injury in N and J Substrains with Adriamycin-Induced Nephropathy. **(A–D)** Immunofluorescence staining and evaluation of glomeruli and podocytes with synaptopodin, WT1 and nephrin (scale bar = 20 μm). Representative images showing glomerular and podocyte **(i)** SYNPO (green)/DAPI (blue), **(ii)** WT1 (green), **(iii)** WT1 (green)/DAPI (blue), and **(iv)** NPHS1 (green)/DAPI (blue) staining from **(A,B)** C57BL/6 N control and ADR-injected mice, and **(C,D)**, C57BL/6 J control and ADR-injected mice. **(ii, iii)** WT1 colocalization with DAPI in the nucleus is indicated by a light blue color. Podocyte and glomerular specific staining is indicated with arrows. At least three glomeruli from three mice per group were examined. **(E)** RNA was isolated from the kidney cortex from C57BL/6 J and N substrains, control and ADR-injected mice. *Nphs2*/Podocin expression fold change is shown comparing control and ADR-treated mice. ***p < 0.001 by unpaired student *t*-test, *n* = 3/group.

The structural and functional integrity of podocytes, specialized terminally differentiated epithelial cells in the glomeruli, is crucial for the optimum functioning of the glomerular filtration barrier (Benzing, 2020). Synaptopodin is one of the key determinants of podocyte actin cytoskeletal integrity, nephrin and podocin form the crucial components of the slit diaphragm and WT-1 is an important transcriptional master regulator of podocyte function (Asanuma et al., 2005; Morrison et al., 2008; Benzing, 2020). We evaluated the expression of these critical podocyte markers in the kidneys of both N and J substrains of control and ADR-injured mice and found all these markers to be downregulated with ADR injury, and these effects were more pronounced in the N substrain than the J substrain (Figures 3A–E). Synpatopodin expression was diminished and disrupted in the injured glomeruli in both the substrains, with higher reduction observed in the N substrain than the J substrain. WT1 expression was found to be nuclear in healthy podocytes and the staining of nuclear WT1 in the podocytes was reduced in ADR-injured glomeruli in both N and J substrains. Although we observed some background non-specific staining with nephrin antibodies, its glomerular specific staining was also reduced in the ADR-injured glomeruli, more so in the N substrain than the J substrain. *Nphs2* encoding for podocin was found to be reduced in the N substrain mice after ADR injury and showed a trend towards reduction in the J substrain.

## Discussion

Mouse models are invaluable resources for reverse genetics to understand pathophysiology and to evaluate therapeutics. Most of the genetic glomerular disease models are generated in the C57BL/6 strain, however this strain has been historically categorized as a resistant strain to ADR-induced nephropathy (Zheng et al., 2005; Zheng et al., 2006; Papeta et al., 2010). A few recent reports have implicated the use of C57BL/6 strain for ADR-induced nephrosis, however the categorization of substrain susceptibilities is unavailable (Jeansson et al., 2009; Heikkila et al., 2010; Lee and Harris, 2011; Hakroush et al., 2014; Arif et al., 2016; Sonneveld et al., 2017; Yang et al., 2018). Furthermore, reports in the literature are somewhat confusing attributable to variability in dosage, timing and frequency of ADR administration. In order to address these and some of our own challenges of availability of genetically modified mice regarding genes of our interest in J and N substrains of C57BL/6 mice, we conducted regimented experimental studies to compare the extent of ADR-induced nephropathy in J and N substrains. We induced nephropathy with 2X ADR injections and measured albuminuria throughout the study for 6 weeks with terminal histological examinations and evaluation of glomerular and podocyte injury. A higher ADR dose and frequency were chosen than the conventional ADR-dosing schedule involving the susceptible BALB/cJ and 129/SvJ strains because of the notion that C57BL/6 mice are resistant strains and what we deduced as optimal for consistent nephropathy outcomes. We were able to successfully develop a robust nephrosis model in the N substrain and a mild nephrosis model in the J substrain.

In the mid-1990s, it became evident that ADR-induced FSGS was highly strain dependent in mice as the mouse strains BALB/cJ and 129/SvJ were found to be highly susceptible, while C57BL/6 and FVB/NJ were deemed to be resistant (Kimura et al., 1993; Chen et al., 1995; Chen et al., 1998; Wang et al., 2000; Pippin et al., 2009). This has been a roadblock to utilize the C57BL/6 strain as a model of FSGS. In our own previously reported study (Nie et al., 2018), significant amount of time and resources were utilized in backcrossing the C57BL/6 mice harboring knock-out of our genes of interest (MK2 and MK3) to the 129/SvJ strain to render them susceptible to ADR-nephrosis. Such experiments can benefit from the knowledge of difference in substrain susceptibility between N and J and the ability to even make the J substrain modestly proteinuric with a modified protocol, as is described in this study. In some cases, it might be even preferable to utilize the C57BL/6 strain over the sensitive BALB/c mice, which respond poorly to kidney perfusion, thus not allowing for complete study of disease progression (Jeansson et al., 2009). With increasing complexities in mouse genetics and breeding experiments, monitoring of genetically engineered mice even within the C57BL/6 background remain to be challenging. The N substrain is a National Institute of Health (NIH) subline of C57BL/6, which was separated from C57BL/6J (Jackson Laboratories) in 1951, and it can be distinguished from C57BL/6J with at least 11 SNP differences, which likely results in phenotypic differences affecting a broad range of areas including metabolism, immune function, and cardiovascular function (Simon et al., 2013; Fontaine and Davis, 2016). The current study highlights the usefulness of both C57BL/6 substrains for ADR-induced nephrosis by clarifying the susceptibility differences in the magnitude of ADR-induced nephropathy. A recent brief editorial highlighted the importance of discriminating between these two substrains in the context of ADR-induced nephrosis (Arif et al., 2016). Our study has further delved into this critical observation to demonstrate a robust vs. modest induction of ADR-induced nephropathy or FSGS in N vs. J mice, respectively, with a standardized protocol. Furthermore, our findings demonstrate a robust induction of proteinuria in N substrain as opposed to what was reported as just a mild proteinuria in the N substrain. Second, we also demonstrate a modest, but significant induction of proteinuria and glomerular lesions in J substrain, while many previous reports just dismissed this substrain to be completely resistant with absence of any proteinuria. Our study was also different in other aspects, such as utilization of 2 doses of ADR vs. one dose of ADR, serial albuminuria results shown until week 6 vs. only one time point 4 weeks albuminuria depiction, route of ADR administration (i.v. vs. retroorbital), and glomerular histology results vs. only foot process effacement (Arif et al., 2016), which is only an indicator of a less severe than FSGS form of NS (i.e., minimal change disease). Moreover, no detailed histological alterations were previously examined for ADR treated N and J substrains, It is important to note that we have observed glomerular and tubular histological lesions in N and mild alterations in J substrain, which were found to be correlative with proteinuria in the respective substrains. Furthermore, we have observed reduction in the expression of critical podocyte markers, synaptopodin, nephrin, WT1 and podocin, implicating podocyte damage and injury in both substrains with ADR-nephrosis, with more pronounced reduction in the N substrain compared to the J substrain. These changes in podocyte markers are consistent with the occurrence of proteinuria, which further underscores the importance of these models as relevant systems to evaluate podocyte-specific gene knock outs and podocyte-targeted therapies for FSGS.

We anticipate our findings to be valuable for the nephrology community in planning mouse studies to recapitulate FSGS. First, it is important to understand the substrain differences of J and N if the gene of interest is modified in the C57BL/6 background. This would be particularly beneficial in the N background before spending time and resources to backcross to a more susceptible strain such as BALB/cJ or 129/SvJ. This is also relevant as backcrossing into a different strain often leads to residual differences in the genetic background, if one compares the knock-out on the backcrossed strain with the wildtype of that strain (Hay et al., 2021). Second, the model of modest proteinuria and nephropathy in the J substrain could potentially be a favorable model for certain reverse genetics, where the expectation is the exacerbation of injury with a double hit or insult such as knock-out or knock-in of relevant genes critical for podocyte function. Consistently, a few studies have shown increase in nephrosis just after 3 weeks with e.g., podocyte-specific deletion of PPARγ when challenged with ADR in mice on C57BL/6J background (Jeansson et al., 2009; Heikkila et al., 2010; Hakroush et al., 2014; Sonneveld et al., 2017). Third, our studies suggest that it is critical to follow with a mandatory second dose of ADR at 15 mg/kg to induce nephropathy and persistent proteinuria. Fourth, it is important to relieve the distress caused by a strong drug such as ADR to the mice at multiple organ level by administering the minimal dose required and providing pain relief measures and hydration imparting nutragels during the experiment. Depending on the dosage and strain, mortality and severe weight loss are common problems associated with ADR-induced nephrosis even in susceptible strains such as 129/SvJ and BALB/cJ, which causes much distress to the animals and hinders the experiments and skews the results (Pippin et al., 2009; Hakroush et al., 2014; Nie et al., 2018). We have successfully induced nephrosis while observing 0% mortality with simple palliative measures, in both J and N substrains of C57BL/6. Only male mice were used in this study as the female mice are known to be more resistant to ADR-induced injury, especially in C57BL/6 J strain (Lee and Harris, 2011; Grant et al., 2019), although future studies may shed more light on the sex-dependent outcomes.

In summary, our studies demonstrate a reproducible method to induce robust and modest nephrosis in the N and J substrains of C57BL/6 mice, respectively, using ADR. This enables further studies, especially proper selection of C57BL/6 substrains for genetic manipulations to study ADR and relevant studies in established knockout or genetically manipulated animal models where the strain or substrain were predetermined. We also believe these results will persuade the scientific community to consider C57BL/6 mouse substrains J and N as distinctly useful models for the studies of kidney injuries.

## Data Availability

The original contributions presented in the study are included in the article/Supplementary Material, further inquiries can be directed to the corresponding author.
